# Delayed-Onset Hematomas Following Mohs Surgery in Patients Taking a Direct Oral Anticoagulant

**DOI:** 10.7759/cureus.83408

**Published:** 2025-05-03

**Authors:** Monica Constantinescu, Olivia Penela, Chinmoy Bhate, Armand Cognetta

**Affiliations:** 1 Dermatology, Dermatology Associates of Tallahassee, Tallahassee, USA; 2 Medicine, Mercer University School of Medicine, Columbus, USA; 3 Dermatology, Florida State University College of Medicine, Tallahassee, USA

**Keywords:** apixaban, blood thinner, factor xa inhibitor, hematoma, mohs surgery

## Abstract

Direct factor Xa inhibitors or direct oral anticoagulants, like apixaban, are used to reduce the risk of stroke, deep vein thrombosis, and pulmonary embolism. It is essential that patients prescribed these drugs follow the instructions carefully. When a patient needs surgery and is on a direct oral anticoagulant, the question arises as to whether they should stop taking it before the elective surgery. There are currently no well-established guidelines instructing whether a patient should discontinue taking them before a Mohs procedure. A Mohs procedure is a precise surgery that removes skin cancers. We describe two cases of delayed-onset hematoma formation following Mohs micrographic surgery and reconstruction in patients taking direct oral anticoagulants not withheld prior to the procedure. Due to the possible uniqueness of late-onset hematoma formation greater than two weeks post-operatively in patients taking apixaban, we wanted to bring this event to the attention of other physicians. This report highlights the need to instruct patients on the possible complications of continuing the prescription and the importance of consulting the prescribing doctor before telling the patient to cease taking the drugs.

## Introduction

Apixaban is a direct factor Xa inhibitor approved for clinical use in reducing stroke risk in non-valvular atrial fibrillation and treatment and prevention of deep vein thrombosis or pulmonary embolism [1]. Like other newer blood-thinning agents, it is considered a direct oral anticoagulant (DOAC) and is metabolized mainly by the CYP3A4 enzyme that is present in the liver and intestines [1]. This drug is eliminated in both urine and feces, with renal excretion accounting for about 27% of the elimination [1]. Apixaban is not known to have interactions with other commonly prescribed medications, but the dosage may need to be decreased by 50% if the patient is over 80 years old, under 60 kg, or has a creatinine clearance below 30 mL/min [1]. The FDA package insert advises that this drug be avoided in patients with severe hepatic impairment [1]. Unlike traditional anticoagulants, such as warfarin, it is typically prescribed at a fixed twice-daily regimen without the need for monitoring. Its peak effects occur approximately 1 to 2 hours post-oral dose, and its half-life is 12 hours [1]. Dermatologic guidelines typically do not advocate halting prescribed blood thinning medications prior to cutaneous surgery, including Mohs micrographic surgery (MMS) and cutaneous reconstruction [2,3]. These guidelines have been updated to include the DOACs and stated that those who stop taking their DOAC do not have a decreased risk of hemorrhagic complications compared to those who continue taking them [4]. According to the British Society of Dermatological Surgery, there may be some scenarios where stopping DOACs perioperatively is advisable [5]. These guidelines specifically state that the physician doing the procedure should weigh the risks/benefits [5]. Although most guidelines do not advocate for stopping these drugs, some physicians may take different approaches with their patients. Some may consult the prescribing physician before making the decision to stop the medication, or others may just proceed without altering the medication. Risks of morbidity and/or mortality associated with thrombosis, stroke, or myocardial infarction are weighed against the risk of cutaneous bleeding [2]. The specific type of cutaneous bleeding we are focused on is hematoma formation. Hematomas are closed wounds where blood collects and fills the space because it has nowhere to drain [6]. There are multiple types of hematomas, and they are usually defined by where they present [6]. For example, the hematomas we are focusing on are called subcutaneous hematomas, which present in the skin [6].

Subcutaneous hematomas can cause longer healing times, less cosmetically pleasing scars, and possibly require additional intervention from the surgeon. There are also other types, such as subdural hematomas, which occur within the skull; rectus sheath hematomas, located in the abdominal wall; and subungual hematomas, which form beneath the nails [6]. For a normal Mohs procedure, local anesthetic is used to numb the area, and the skin cancer is removed. Once completely removed, the wound is then sutured closed. The current guidelines for preventing post-operative complications advise patients to refrain from strenuous physical activity for a week, keep Vaseline on the wound until the sutures are removed, and apply ice packs regularly during the day to reduce swelling [7]. Although most post-operative bleeding events occur within the first 24 to 48 hours, we herein describe two cases of delayed-onset hematoma formation over two weeks after MMS in patients treated with apixaban.

## Case presentation

**Figure 1:**
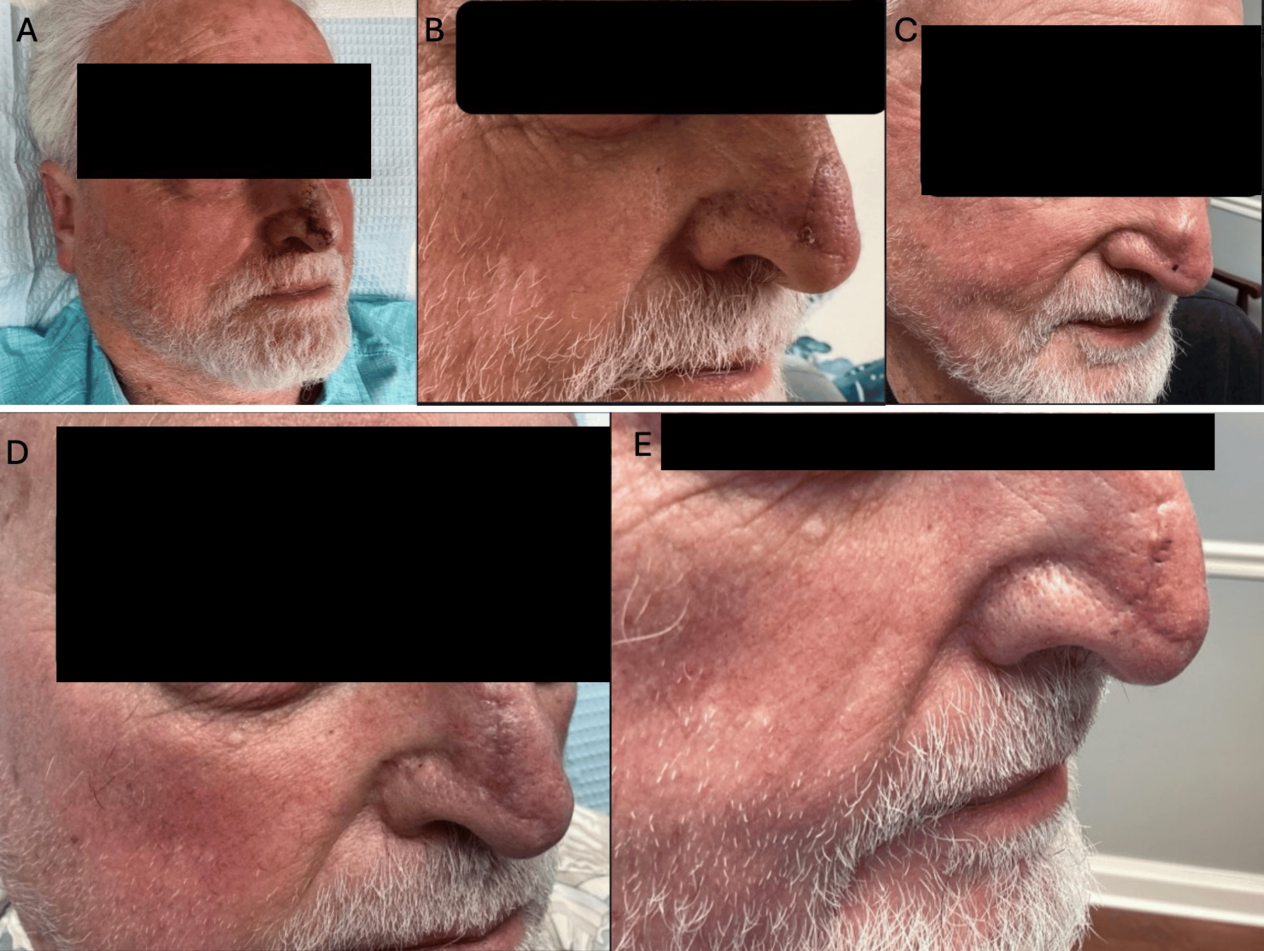
Patient progression during the course of treatment. (A) Sutured repair of advancement flap of the nasal tip. (B) Healing incision three weeks post-procedure. (C) Delayed onset of hematoma four months after Mohs surgery. (D) Healing incision two weeks after excision of hematoma. (E) Patient 235 days post-procedure.

An 83-year-old White male with atrial fibrillation treated with apixaban (Eliquis), 5 mg daily, underwent single-stage MMS for treatment of a nodular basal cell carcinoma on the nasal supratip. The defect was repaired with a local advancement flap without complication, using 5-0 poliglecaprone 25 subcutaneous and dermal sutures, and 6-0 polypropylene running epidermal sutures (Figures 1A, 1B). His recovery was unremarkable until four months post-operatively, when he noticed persistent swelling at the surgical site (Figure 1C). This was evaluated and found to be a hematoma, which was incised and drained but subsequently reaccumulated. One month later, the organized hematoma was excised without complication (Figure 1D). The patient was seen after the excision and was found to be fully healed (Figure 1E).

**Figure 2:**
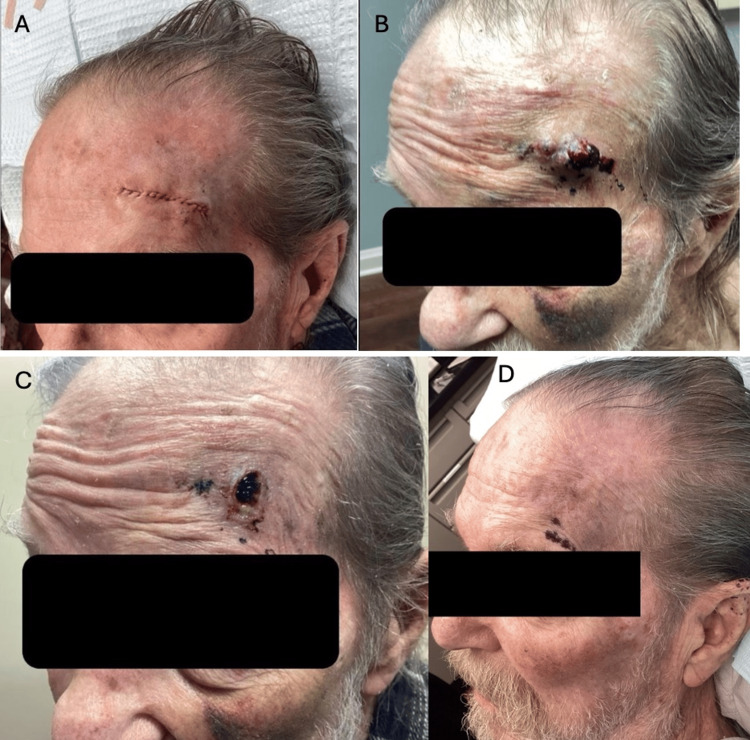
Treatment timeline of the patient. (A) Sutured repair of linear wound on lateral forehead. (B) Delayed onset hematoma. (C) Delayed hematoma 12 days after evacuation. (D) Patient 128 days post original procedure.

An 86-year-old White male with atrial fibrillation, also taking 5 mg apixaban daily, underwent single-stage MMS for a squamous cell carcinoma in situ on the left lateral forehead. The defect was repaired with an intermediate linear closure using 4-0 poliglecaprone 25 subcutaneous and dermal sutures, and 5-0 plain gut running and interrupted epidermal sutures (Figure 2A). Approximately two weeks after the procedure, the patient noted bleeding from the surgical wound and the formation of a 1.4 x 1.0 cm ecchymotic dermal nodule (Figure 2B). A shave biopsy at that time revealed an ulcerated hematoma, and the wound healed by secondary intention (Figure 2C). The patient was observed to have a fully healed hematoma 128 days after the original procedure (Figure 2D).

## Discussion

Post-operative bleeding is a common complication of minor skin surgery. Hematoma formation is more uncommon, occurring in only 0.1-2.4% of dermatological procedures and is not life-threatening [8]. It is typically controlled with caustic or non-caustic topical hemostatic agents, suture, cautery or electrosurgery, or direct pressure with dressings [9]. Other possible complications of Mohs surgery are post-operative hemorrhage, wound dehiscence, wound infection, flap necrosis, and skin graft necrosis [10]. Most guidelines do not advocate for the cessation of prescribed blood-thinning medications prior to cutaneous surgery, as the benefit typically outweighs the risk of thrombosis [9]. Although we focus in this case study on DOACs as the possible cause of the hematomas, it is worth noting that there are other possible causes of complications and the possibility that the hematomas could have formed if the drugs were discontinued perioperatively. For example, patients with medical comorbidities like coagulation factor deficiencies can increase their likelihood of having a bleeding complication from surgery [11].

Direct oral anticoagulants inhibit proteins within the coagulation cascade. Two main categories exist as follows: direct thrombin inhibitors, such as dabigatran, and factor Xa inhibitors, such as apixaban [12]. Both types bind reversibly to their respective targets [13]. Apixaban was specifically found to be superior to warfarin in both safety and efficacy in reducing strokes in patients with atrial fibrillation [13].

Spontaneous soft tissue hematoma formation has been described in patients taking apixaban. One elderly female patient taking apixaban for atrial fibrillation presented with abdominal pain and cough and was found to have a spontaneous rectus sheath hematoma in one report [14]. This patient developed the hematoma three months after starting apixaban with no other lifestyle modifications in that timeframe. Another elderly patient presented with a hematoma of the right anterolateral abdominal wall three days after the initiation of low molecular weight heparin therapy [15]. Acute compartment syndrome secondary to a spontaneous hematoma has also been described in a patient taking apixaban [16]. This patient started taking the drug eight months prior due to a past deep vein thrombosis [16]. Additionally, delayed onset intracranial hematomas have been described in the emergency medicine literature following head trauma [17,18]. Some patients had spontaneous epidural hematoma formation one month after starting the drug, and others had upper limb hematoma formation six days after a small fall [19,20]. With these other presentations, it is difficult to discern whether just the DOAC is causing the hematoma formation or other factors, since these hematomas formed at different times and in different places in each patient. Due to the broad presentations in these cases, it is important to think thoroughly about all the possible mechanisms for the formation of the different hematomas. They could have happened not only because of the drugs' pharmacology but also due to personal factors like differences in patient history. For example, a couple of the patients had hematoma formation after a surgical procedure, while others had them after trauma or spontaneously.

In our practice, we include delayed hematoma in the informed consent as a possible complication of Mohs surgery. As specific protocols or expert consensus guidelines for the perioperative management of DOACs in dermatologic surgery are lacking, the Mohs surgeon decided that the risk of stopping the medication was greater than the risk of bleeding in these cases. It is now our practice to inform the prescribing doctor (primary care physician or cardiologist) of upcoming Mohs procedures and to seek their guidance regarding perioperative dosing of DOACs based on the patient’s relevant medical comorbidities and overall risk of a thrombotic event.

## Conclusions

This study highlighted two cases of spontaneous hematoma formation after Mohs surgery in patients taking direct oral anticoagulant therapy. Although these complications happened to arise in patients both taking apixaban, it is possible that they would have formed anyway with the discontinuation of the medication. If the medication was discontinued, hematoma formation might have occurred anyway because the patient would have resumed taking apixaban before the time of hematoma formation. Due to the limitations of the present case study, we cannot prove cause and effect just from these two cases; however, we want to bring focus to the discussed possible adverse complications. Since there is a lack of literature on this subject, this report concluded the importance of informing the prescribing doctor of the surgery and seeking direct guidance on whether to inform the patient to refrain from taking the medication before the procedure. There is a need for further research to draw stronger conclusions on the incidence and risk factors of delayed-onset hematoma formation in this patient population.
